# Metagenomes, metatranscriptomes and microbiomes of naturally decomposing deadwood

**DOI:** 10.1038/s41597-021-00987-8

**Published:** 2021-08-03

**Authors:** Vojtěch Tláskal, Vendula Brabcová, Tomáš Větrovský, Rubén López-Mondéjar, Lummy Maria Oliveira Monteiro, João Pedro Saraiva, Ulisses Nunes da Rocha, Petr Baldrian

**Affiliations:** 1 Laboratory of Environmental Microbiology, Institute of Microbiology of the Czech Academy of Sciences, Videnska 1083, 14220 Praha 4, Czech Republic; 2 Department of Environmental Microbiology, Helmholtz Centre for Environmental Research – UFZ, Permoserstraße 15, 04318 Leipzig, Germany

**Keywords:** Forest ecology, Environmental microbiology

## Abstract

Deadwood represents significant carbon (C) stock in a temperate forests. Its decomposition and C mobilization is accomplished by decomposer microorganisms – fungi and bacteria – who also supply the foodweb of commensalist microbes. Due to the ecosystem-level importance of deadwood habitat as a C and nutrient stock with significant nitrogen fixation, the deadwood microbiome composition and function are critical to understanding the microbial processes related to its decomposition. We present a comprehensive suite of data packages obtained through environmental DNA and RNA sequencing from natural deadwood. Data provide a complex picture of the composition and function of microbiome on decomposing trunks of European beech (*Fagus sylvatica* L.) in a natural forest. Packages include deadwood metagenomes, metatranscriptomes, sequences of total RNA, bacterial genomes resolved from metagenomic data and the 16S rRNA gene and ITS2 metabarcoding markers to characterize the bacterial and fungal communities. This project will be of use to microbiologists, environmental biologists and biogeochemists interested in the microbial processes associated with the transformation of recalcitrant plant biomass.

## Abstract (summary)

**Table:** 

Measurement(s)	metagenomic data • metatranscriptomic data • microbiome • RNA-seq of total RNA
Technology Type(s)	DNA sequencing • RNA-seq of total RNA • amplicon sequencing • RNA sequencing
Factor Type(s)	time of decomposition
Sample Characteristic - Organism	Fungi • Bacteria
Sample Characteristic - Environment	wood
Sample Characteristic - Location	Narodni prirodni rezervace Zofinsky prales

Machine-accessible metadata file describing the reported data: 10.6084/m9.figshare.14821752

## Background & Summary

Forests, and especially unmanaged natural forests, accumulate and store large amounts of carbon (C)^1^. A substantial fraction of this C stock – 73 ± 6 Pg or 8% of the total global forest C stock is contained within deadwood^2^. This C pool is transient because during its transformation by saprotrophic organisms most C is liberated as CO_2_ into the atmosphere^3^, while the rest is sequestered in soils as dissolved organic C, within microbial biomass or as a part of the soil organic matter – along with other nutrients^4^. Fungi in deadwood appear to be major decomposers using extracellular enzymes for the decomposition of recalcitrant plant biopolymers as shown in associated study^5^. Fungi also determine the bacterial community composition^6,7^. Bacterial fixation of atmospheric N_2_ was shown to substantially contribute to the nitrogen (N) increase in deadwood during decomposition^5,8,9^. In addition to bacteria and fungi, deadwood also hosts a suite of other organisms including archaea, viruses, protists, nematodes and insects, whose roles in deadwood are so far unknown. In order to understand the deadwood as a dynamic habitat, it is necessary to describe the composition of associated microorganisms with an emphasis on the major groups – fungi and bacteria – whose ecologies are often genus-specific^10^. Further, it is important to link deadwood-associated organisms to processes occurring at different stages of decomposition either by characterization of isolates^7,11^ or by cultivation-independent techniques.

**Fig. 1:**
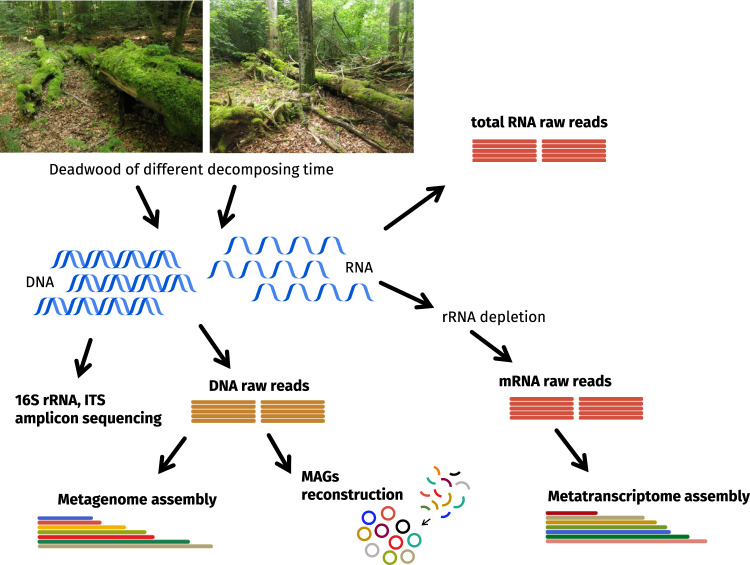
Study workflow and sequencing data sources. Available data packages are in bold. The age class 1 was <4 years since tree death, class 2 4–7 years, class 3 8–19 years, class 4 20–41 years and class 5 > 41 years (n = 5 per age class).

**Fig. 2:**
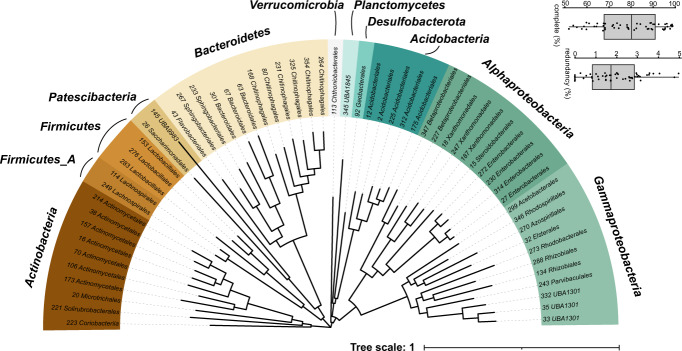
Phylogenetic tree of 58 high-quality MAGs based on set of bacterial single copy genes. Phyla (or classes of *Proteobacteria*) are color-coded, tree tips are labelled with order taxonomy obtained from GTDB database. Boxplots represent completeness and redundancy values of MAGs.

In this Data Descriptor we present the comprehensive datasets of DNA and RNA-derived data and sample metadata to characterize deadwood organisms and their activity at various stages of decomposition (Table 1, Supplementary Table 1). The data derived from DNA representing the community composition and genomic potential, include 16S rRNA gene sequences and ITS2 sequences, metagenomics reads, metagenome assembly and bacterial metagenome-assembled genomes (MAGs^12^). The data derived from RNA are represented by the total RNA reads whose majority originates from ribosomal RNA and which are taxonomically assignable and thus can be used as a proxy for the PCR-unbiased view of community composition. Further, data contain metatranscriptome raw reads and assembly that represent the processes occurring in deadwood. The dataset characterizes decomposing trunks of the European beech (*Fagus sylvatica* L.) in a beech-dominated natural forest in the temperate Europe (Fig. 1). The metagenome was assembled from 25 DNA samples of deadwood with decomposition time ranging from young wood (<4 years since tree death) to almost completely decomposed wood (>41 years of decomposition). It was possible to perform the resolving of 58 high-quality metagenome assembled genomes (MAGs) with a total of 19.5 × 10^3^ contigs spanning 10 bacterial phyla including those that are difficult to culture, such as *Acidobacteria*, *Patescibacteria*, *Verrucomicrobia* and *Planctomycetes* (Fig. 2, Supplementary Table 2). 16S rRNA gene and ITS2 amplicon data contribute to comparison of microbial diversity and occurrence patterns at the global scale using public databases GlobalFungi^13^ or Earth Microbiome^14^ (Supplementary Fig. 1). Deadwood metatranscriptome was assembled from 10 RNA samples spanning two age classes of decomposing deadwood (between 4 and 19 years old). The amount of raw and assembled data in individual data packages is summarised in the Table 2. Overview of data previously used to describe complementarity of fungal and bacterial roles in deadwood is specified in Data Records summary^5^.

**Table 1:** Sample metadata for 25 samples used for sequencing.

Sample	BioSample	Location	time since death (years)	diameter (cm)	length (m)	pH	N content (%)	C content (%)	water content (%)	nucleic acids extraction
sample_130	SAMN13925149	48.666701 N 14.704277 E	<4	50	29.4	5.4	0.28	49.09	40.9	DNA
sample_131	SAMN13925150	48.666134 N 14.707596 E	<4	70	24.3	5.46	0.19	50.06	37.4	DNA
sample_132	SAMN13925151	48.667505 N 14.707659 E	<4	94	37.1	4.65	0.27	49.7	36.1	DNA
sample_133	SAMN13925152	48.664471 N 14.709305 E	<4	62	22.8	5.33	0.19	49.88	42.5	DNA
sample_134	SAMN13925153	48.66419 N 14.705787 E	<4	60	18.6	4.96	0.42	48.55	49.3	DNA
sample_006	SAMN13925154	48.666377 N 14.709879 E	4–7	60	8.4	3.95	0.71	50.65	83.1	DNA, RNA
sample_007	SAMN13762420	48.666311 N 14.709489 E	4–7	42	9.8	4.76	0.12	22.45	47.5	DNA, RNA
sample_044	SAMN13925156	48.66597 N 14.706626 E	4–7	55	9.7	3.87	0.43	50.29	49.0	DNA, RNA
sample_110	SAMN13925157	48.665322 N 14.708804 E	4–7	65	7.7	4.12	0.68	49.62	75.2	DNA, RNA
sample_116	SAMN13925158	48.666959 N 14.704827 E	4–7	85	18.1	3.32	0.69	51.25	76.3	DNA, RNA
sample_031	SAMN13925159	48.666891 N 14.703544 E	8–19	95	37.1	4.87	0.34	48.7	52.8	DNA, RNA
sample_049	SAMN13925160	48.665518 N 14.706881 E	8–19	34	20.6	4.44	0.23	49.25	51.8	DNA, RNA
sample_055	SAMN13925161	48.665975 N 14.709225 E	8–19	70	4.0	3.99	0.33	49.93	63.7	DNA, RNA
sample_069	SAMN13925162	48.664308 N 14.704474 E	8–19	75	34.7	4.78	0.5	48.16	82.1	DNA, RNA
sample_106	SAMN13925163	48.664703 N 14.708727 E	8–19	30	22.0	4.07	0.4	49.19	78.1	DNA, RNA
sample_003	SAMN13925164	48.666874 N 14.709428 E	20–41	30	10.8	3.53	0.61	50.23	71.8	DNA
sample_028	SAMN13925165	48.667629 N 14.703671 E	20–41	70	11.5	3.75	2.11	50.83	69.2	DNA
sample_039	SAMN13925166	48.665832 N 14.705361 E	20–41	50	18.6	3.73	1.47	49.96	88.1	DNA
sample_057	SAMN13925167	48.664869 N 14.709048 E	20–41	100	26.2	3.79	0.83	51.07	69.9	DNA
sample_084	SAMN13925168	48.663645 N 14.70741 E	20–41	80	10.9	3.05	1.13	51.31	75.3	DNA
sample_058	SAMN13925169	48.664099 N 14.709529 E	>41	70	11.1	4.15	1.31	56.11	66.2	DNA
sample_101	SAMN13925170	48.66386 N 14.705644 E	>41	70	7.8	3.34	1.95	51.7	83.9	DNA
sample_111	SAMN13925171	48.667297 N 14.704368 E	>41	48	17.5	4.35	0.51	54.92	72.2	DNA
sample_113	SAMN13925172	48.666702 N 14.707763 E	>41	80	8.5	3.61	1.47	52.2	75.4	DNA
sample_115	SAMN13925173	48.667087 N 14.705332 E	>41	60	10.7	3.33	0.67	50.9	64.6	DNA

**Table 2:** Raw read counts in individual data packages (mean ± SE) and statistics of metagenome and metatranscriptome assembly.

data type	N samples	raw reads	common assembly [contigs]	L50 [contigs]
metagenome	25	22.5 ± 7.2 × 10^6^	17.9 × 10^6^	4.5 × 10^6^
metatranscriptome	10	31.3 ± 9.1 × 10^6^	1.3 × 10^6^	0.5 × 10^6^
total RNA	10	15.1 ± 1.8 × 10^6^		
ITS2 marker	25	18.3 ± 1.4 × 10^3^		
16S rRNA marker	25	21.7 ± 1.6 × 10^3^		

The previous studies devoted to deadwood have not seen such a comprehensive set of information about the associated biota. These data significantly improve the width and resolution and thus the understanding of the biodiversity of deadwood associated biota and its function. Given that natural forests represent an essential ecosystem concerning C storage and nutrient cycling, the data within this Data Descriptor make it possible to fully appreciate the ecosystem-level roles that deadwood plays in forest ecosystems.

## Methods

### Study area and sampling

Deadwood was sampled in the core zone of the Žofínský prales National Nature Reserve, an unmanaged forest in the south of the Czech Republic (48°39′57″N, 14°42′24″E) as described earlier in the associated study^5^. The core zone had never been managed and any human intervention stopped in 1838 when it was declared as reserve. It thus represents a rare fragment of European temperate virgin forest left to spontaneous development. The reserve is situated at 730–830 m a.s.l., bedrock is almost homogeneous and consists of finely to medium-grainy porphyritic and biotite granite. Annual average rainfall is 866 mm and annual average temperature is 6.2 °C^15^.

Previous analysis indicated that deadwood age (time of decomposition) significantly affects both wood chemistry and the composition of microbial communities^16,17^. We thus randomly selected dead tree trunks that represented age classes 1–5 assigned based on the decomposition length^18^. Each age class was represented by five logs of 30–100 cm diameter (Table 1). The age class 1 was <4 years since tree death, class 2 4–7 years, class 3 8–19 years, class 4 20–41 years and class 5 > 41 years (n = 5 per age class); only trees that were not alive and not decomposed before downing were considered. DNA was extracted from all logs. Due to sample-specific RNA extraction yields, RNA of sufficient amount and quality was extracted from the subset of logs (age classes 2 and 3). Sampling was performed in November 2016. The length of each selected log (or the sum of the lengths of its fragments) was measured and four samples were collected at the positions of 20%, 40%, 60% and 80% of the log length by drilling. This was performed vertically from the middle of the upper surface through the whole diameter using an electric drill with an auger diameter of 10 mm. The sawdust from all four drill holes from each log was pooled and immediately frozen using liquid nitrogen, transported to the laboratory on dry ice and stored at −80 °C until further processing.

### Sample processing, DNA and RNA extraction

Sample characteristics as pH, C, N and water content were measured as described in the associated study^5^. Similarly, workflow of nucleic acid preparation, ligation and sequencing was described previously. Briefly, wood samples (approximately 10 g of material) were homogenized using a mortar and pestle under liquid nitrogen prior to nucleic acid extraction and thoroughly mixed. Total DNA was extracted in triplicate from 200 mg batches of finely ground wood powder using a NucleoSpin Soil kit (Macherey-Nagel).

Total RNA was extracted in triplicate from 200 mg batches of sample using NucleoSpin RNA Plant kit (Macherey-Nagel) according to manufacturer’s protocol after mixing with 900 μl of the RA1 buffer and shaking on FastPrep-24 (MP Biomedicals) at 6.5 ms^−1^ twice for 20 s. Triplicates were pooled and treated with OneStep PCR Inhibitor Removal kit (Zymo Research), DNA was removed using DNA-free DNA Removal Kit (Thermo Fisher Scientific). The efficiency of DNA removal was confirmed by the negative PCR results with the bacterial primers 515F and 806R^19^. RNA quality was assessed using a 2100 Bioanalyzer (Agilent Technologies).

### Analysis of deadwood-associated organisms

To estimate the relative representation of deadwood-associated organisms in deadwood, total RNA was sequenced since the majority of the RNA represents either small subunit ribosomal RNA or large subunits ribosomal RNA that allows the identification of organisms by BLASTing against the curated databases from SILVA^20,21^. Read abundances represent the abundances of ribosomes of each taxon and thus reflect the abundance of each taxon. Libraries for high-throughput sequencing of total RNA were prepared using TruSeq RNA Sample Prep Kit v2 (Illumina) according to the manufacturer’s instructions, omitting the initial capture of polyA tails to enable total RNA to be ligated. Samples were pooled in equimolar volumes and sequenced on an Illumina HiSeq 2500 (2 × 250 bases) at Brigham Young University Sequencing Centre, USA.

### Metatranscriptomics and metagenomics

For metatranscriptome analysis, the content of rRNA in RNA samples was reduced as described previously^5,22^ using a combination of Ribo-Zero rRNA Removal Kit Human/Mouse/Rat and Ribo-Zero rRNA Removal Kit Bacteria (Illumina). Oligonucleotide probes from both types of Ribo-Zero kits were mixed together and added to each sample which allowed their annealing to rRNA and subsequent rRNA removal. The efficiency of the removal was checked using a 2100 Bioanalyzer and removal was repeated when necessary. Reverse transcription was performed with SuperScript III (Thermo Fisher Scientific). Libraries for high throughput sequencing were prepared using the ScriptSeq v2 RNA-Seq Library Preparation Kit (Illumina) according to the manufacturer’s instructions with a final 14 cycles of amplification by FailSafe PCR Enzyme (Lucigen).

The NEBNext Ultra II DNA Library Prep Kit for Illumina (New England BioLabs) was used to generate metagenome libraries according to the manufacturer’s instructions. Samples of the metagenome and metatranscriptome were pooled in equimolar volumes and sequenced on an Illumina HiSeq 2500 (2 × 250 bases) at Brigham Young University Sequencing Centre, USA.

Metagenome assembly and annotation were performed as described previously^5^. Briefly, Trimmomatic 0.36^23^ and FASTX-Toolkit (http://hannonlab.cshl.edu/fastx_toolkit/) were used to remove adaptor contamination, trim low-quality ends of reads and omit reads with overall low quality (<30), sequences shorter than 50 bp were omitted. Combined assembly of all 25 samples was performed using MEGAHIT 1.1.3^24^. Metagenome sequencing yielded on average 22.5 ± 7.2 million reads per sample that were assembled into 17,936,557 contigs over 200 bp in length.

Metatranscriptome (MT) assembly and annotation were performed as described previously^5^. Trimmomatic 0.36^23^ and FASTX-Toolkit (http://hannonlab.cshl.edu/fastx_toolkit/) were used to remove adaptor contamination, trim low-quality ends of reads and omit reads with overall low quality (<30), sequences shorter than 50 bp were omitted. mRNA reads were filtered from the files using the bbduk.sh 38.26 program in BBTools (https://sourceforge.net/projects/bbmap/). Combined assembly was performed using MEGAHIT 1.1.3^24^. Metatranscriptome sequencing yielded on average 31.3 ± 9.1 million reads per sample that were assembled into 1,332,519 contigs over 200 bp in length.

### Identification and analysis of metagenome-assembled genomes

Bins that represent prokaryotic taxa present in the metagenome were constructed using MetaBAT2^25^ as described previously^5^ with default settings except for the minimal length of contigs set to 2000 bp, which produced bins with overall better statistics than the minimal 2500 bp size. CheckM 1.0.11^26^ served for assigning taxonomy and statistics to bins with *lineage_wf* pipeline. Bins with a completeness score greater than 50% were selected for quality improvement using RefineM according to the instructions of the developers^27^. Briefly, scaffolds with genomic properties (GC content, coverage profiles and tetranucleotide signatures) whose values were different from those expected in each bin were excluded. These values were calculated based on the mean absolute error and correlation criteria. Next, the refined bins were further processed to identify and remove scaffolds with taxonomic assignments different from those assigned to the bin. Lastly, the scaffolds that possessed 16S rRNA genes divergent from the taxonomic affiliation of the refined bins were removed. The taxonomy of the bins was inferred by GTDB-Tk^28^. 58 bins with quality scores >50 (CheckM completeness value - 5 × redundancy value) were considered metagenome-assembled genomes (MAGs) as defined by^27^ and deposited in the NCBI database.

GToTree v1.5.39^29^ together with Prodigal^30^, HMMER3^31^, Muscle^32^, trimAI^33^, FastTree2^34^ were used to infer phylogeny of MAGs based on set of 74 bacterial single-copy gene HMM profiles with minimal marker share >25%.

### ITS2 and 16S rRNA gene amplicon sequencing and analysis

Subsamples of DNA were used to amplify the fungal ITS2 region using barcoded gITS7 and ITS4 primers^35^ and the hypervariable region V4 of the bacterial 16S rRNA gene using the barcoded primers 515F and 806R^19^ in three PCR reactions per sample. PCR premix for ITS2 or 16S rRNA gene metabarcoding contained 5 μl of 5× Q5 Reaction Buffer, 5 μl of 5× Q5 High GC Enhancer and 0.25 μl Q5 High-Fidelity DNA Polymerase (New England Biolabs), 1.5 μl of BSA (10 mg ml^−1^), 0.5 μl of dNTPs Nucleotide Mix 10 mM (Bioline), 1 μl of each primer, 9.75 μl of H_2_O and 1 μl of template DNA. PCR conditions of fungal amplification were 5 min at 94 °C, 30 cycles of (30 s at 94 °C, 30 s at 56 °C and 30 s 72 °C) and 7 min at 72 °C. PCR conditions of bacterial amplification were 4 min at 94 °C, 25 cycles of (45 s at 94 °C, 60 s at 50 °C and 75 s 72 °C) and 10 min at 72 °C.

Three PCR reactions were pooled together, purified by MinElute PCR Purification Kit (Qiagen) and mixed in equimolar amount according to concentration measured on the Qubit 2.0 Fluorometer (Thermo Fisher Scientific). Sequencing libraries were prepared using the TruSeq PCR-Free Kit (Illumina) according to manufacturer’s instructions and sequencing was performed in-house on Illumina MiSeq (2 × 250 bases).

The amplicon sequencing data were processed using the pipeline SEED 2.1.05^36^. Briefly, paired-end reads were merged using fastq-join^37^. Sequences with ambiguous bases and those with a mean quality score below 30 were omitted. The fungal ITS2 region was extracted using ITS Extractor 1.0.11^38^ before processing. Chimeric sequences were detected using USEARCH 8.1.1861^39^ and deleted, and sequences were clustered using UPARSE implemented within USEARCH^40^ at a 97% similarity level. The most abundant sequences were taken as representative for each OTU. The closest fungal hits at the species level were identified using BLASTn 2.5.0 against UNITE 8.1^41^. Where the best fungal hit showed lower similarity than 97% with 95% coverage, the best genus-level hit was identified. The closest bacterial hit from SILVA SSU database r138^21^ was found by DECIPHER 2.18.1 package^42^ using IDTAXA algorithm with threshold 60^43^. Sequences identified as nonfungal and nonbacterial were discarded.

## Data Records

Data described in this study are summarized in the Supplementary Tables 1 and 2 together with the NCBI accession numbers. Raw sequencing reads (total RNA, metatranscriptomics and metagenomics), assembly files and resolved MAGs have been deposited under NCBI BioProject accession number PRJNA603240^44^. In the associated study^5^ metatranscriptome assembly together with raw reads mapping was used for annotation of microbial functions, total RNA raw reads were used to infer mainly fungal and bacterial taxonomic composition, metagenome assembly and raw reads mapping served solely for MAGs identification. Amplicon data of bacterial 16S rRNA gene and fungal ITS2 that were not published previously, have been deposited under NCBI BioProject accession number PRJNA672674^45^.

## Technical Validation

Deadwood samples were taken aseptically by using sterilized equipment and sterile RNase and DNase-free tubes. RNA and DNA were extracted in an RNase free environment. During the library preparation quantity and quality of the nucleic acids were measured with a Qubit 2.0 Fluorometer and 2100 Bioanalyzer, respectively. PCR with bacterial primers 515F and 806R, negative control containing PCR-grade water and positive control containing extracted bacterial DNA was used to confirm the success of the DNase degradation of RNA samples. 2100 Bioanalyzer measurement was used to confirm successful rRNA depletion. No positive or negative sequencing controls were used to obtain metagenomic and metatranscriptomic data. For 16S rRNA gene amplification, negative and positive controls in the form of PCR-grade water and bacterial DNA, respectively were included. The concentration of the 16S rRNA gene amplicons and controls was measured with a Qubit 2.0 Fluorometer and their quality were analysed using agarose gel electrophoresis. Equimolar pooling of all barcoded sequencing libraries was done according to the quantification using KAPA Library Quantification Kit (Roche).

## Usage Notes

The metagenome and metatranscriptome data described in this Data Descriptor were used to demonstrate the complementarity of fungal and bacterial functions in the carbon and nitrogen cycling in decomposing deadwood and linked them to corresponding biogeochemical processes^5^. However, the analysis on the deposited data packages in the associated study^5^ focused solely on fungi and bacteria despite the presence of other groups of organisms in the studied deadwood. The present deposition of the metagenome assembly and total RNA sequencing data opens the opportunity for biologists interested in virus ecology^46^, bacterial metagenomics^47,48^ and ecology of eukaryota^49,50^ to explore the functional potential of the deadwood-associated biota through the analysis of the metagenome as well as to obtain taxonomic overview of all deadwood-associated organisms using total RNA that allows reliable taxonomic classification of taxa across the whole tree of life^51,52^. Amplicon data described here for the first time offer intra-comparison with metagenomes and metatranscriptomes as well as inter-comparison with further deadwood studies^16,53,54^ and analysis of cross-domain interactions^6,55^. Efforts to collect data and generalize microbial diversity patterns^13,14,56^ profit from fully annotated, accessible and metadata-rich sequences which we present here. The Data Descriptor further provides information for ecologists, biogeochemists and conservation biologists interested in the role of deadwood in ecosystem processes and deadwood associated biodiversity, an important topic of the present research in forest ecology^57^.

## Supplementary information

Supplementary Figure 1

Supplementary Table 1

Supplementary Table 2

## Data Availability

The above methods indicate the programs used for analysis within the relevant sections. The code used to analyse individual data packages is deposited at https://github.com/TlaskalV/Deadwood-microbiome.

## References

[1] Luyssaert S (2008). Old-growth forests as global carbon sinks. Nature.

[2] Pan Y (2011). A large and persistent carbon sink in the world’s forests. Science.

[3] Rinne-Garmston KT (2019). Carbon flux from decomposing wood and its dependency on temperature, wood N_2_ fixation rate, moisture and fungal composition in a Norway spruce forest.. Glob. Chang. Biol..

[4] Šamonil P (2020). Convergence, divergence or chaos? Consequences of tree trunk decay for pedogenesis and the soil microbiome in a temperate natural forest. Geoderma.

[5] Tláskal V (2021). Complementary roles of wood-inhabiting fungi and bacteria facilitate deadwood decomposition. mSystems.

[6] Odriozola I (2021). Fungal communities are important determinants of bacterial community composition in deadwood. mSystems.

[7] Valášková V, de Boer W, Gunnewiek PJAK, Pospíšek M, Baldrian P (2009). Phylogenetic composition and properties of bacteria coexisting with the fungus Hypholoma fasciculare in decaying wood. ISME J..

[8] Brunner A, Kimmins JP (2003). Nitrogen fixation in coarse woody debris of Thuja plicata and Tsuga heterophylla forests on northern Vancouver Island. Can. J. For. Res..

[9] Rinne KT (2016). Accumulation rates and sources of external nitrogen in decaying wood in a Norway spruce dominated forest. Funct. Ecol..

[10] Põlme S (2020). FungalTraits: a user-friendly traits database of fungi and fungus-like stramenopiles. Fungal Divers..

[11] Tláskal V, Baldrian P (2021). Deadwood-inhabiting bacteria show adaptations to changing carbon and nitrogen availability during decomposition. Front. Microbiol..

[12] Lemos LN, Mendes LW, Baldrian P, Pylro VS (2021). Genome-resolved metagenomics is essential for unlocking the microbial black box of the soil. Trends Microbiol..

[13] Větrovský T (2020). GlobalFungi, a global database of fungal occurrences from high-throughput-sequencing metabarcoding studies. Sci. Data.

[14] Thompson LR (2017). A communal catalogue reveals Earth’s multiscale microbial diversity. Nature.

[15] Anderson-Teixeira KJ, Davies SJ, Bennett AC, Muller-landau HC, Wright SJ (2015). CTFS-ForestGEO: a worldwide network monitoring forests in an era of global change. Glob. Chang. Biol..

[16] Baldrian P (2016). Fungi associated with decomposing deadwood in a natural beech-dominated forest. Fungal Ecol..

[17] Smyth CE (2016). Patterns of carbon, nitrogen and phosphorus dynamics in decomposing wood blocks in Canadian forests. Plant Soil.

[18] Král K (2010). Local variability of stand structural features in beech dominated natural forests of Central Europe: Implications for sampling. For. Ecol. Manage..

[19] Caporaso JG (2012). Ultra-high-throughput microbial community analysis on the Illumina HiSeq and MiSeq platforms. ISME J..

[20] Lanzén A (2012). CREST – Classification resources for environmental sequence tags. PLoS One.

[21] Quast C (2013). The SILVA ribosomal RNA gene database project: improved data processing and web-based tools. Nucleic Acids Res..

[22] Žifčáková L, Větrovský T, Howe A, Baldrian P (2016). Microbial activity in forest soil reflects the changes in ecosystem properties between summer and winter. Environ. Microbiol..

[23] Bolger AM, Lohse M, Usadel B (2014). Trimmomatic: A flexible trimmer for Illumina sequence data. Bioinformatics.

[24] Li D, Liu CM, Luo R, Sadakane K, Lam TW (2015). MEGAHIT: An ultra-fast single-node solution for large and complex metagenomics assembly via succinct de Bruijn graph. Bioinformatics.

[25] Kang DD, Froula J, Egan R, Wang Z (2015). MetaBAT, an efficient tool for accurately reconstructing single genomes from complex microbial communities. PeerJ.

[26] Parks DH, Imelfort M, Skennerton CT, Hugenholtz P, Tyson GW (2015). CheckM: assessing the quality of microbial genomes recovered from isolates, single cells, and metagenomes. Genome Res..

[27] Parks DH (2017). Recovery of nearly 8,000 metagenome-assembled genomes substantially expands the tree of life. Nat. Microbiol..

[28] Parks DH (2018). A standardized bacterial taxonomy based on genome phylogeny substantially revises the tree of life. Nat. Biotechnol..

[29] Lee MD (2019). GToTree: A user-friendly workflow for phylogenomics. Bioinformatics.

[30] Hyatt D (2010). Prodigal: prokaryotic gene recognition and translation initiation site identification. BMC Bioinformatics.

[31] Eddy SR (2011). Accelerated profile HMM searches. PLoS Comput. Biol..

[32] Edgar RC (2004). MUSCLE: Multiple sequence alignment with high accuracy and high throughput. Nucleic Acids Res..

[33] Capella-Gutiérrez S, Silla-Martínez JM, Gabaldón T (2009). trimAl: A tool for automated alignment trimming in large-scale phylogenetic analyses. Bioinformatics.

[34] Price MN, Dehal PS, Arkin AP (2010). FastTree 2 – approximately maximum-likelihood trees for large alignments. PLoS One.

[35] Ihrmark K (2012). New primers to amplify the fungal ITS2 region – evaluation by 454-sequencing of artificial and natural communities. FEMS Microbiol. Ecol..

[36] Větrovský T, Baldrian P, Morais D (2018). SEED 2: A user-friendly platform for amplicon high-throughput sequencing data analyses. Bioinformatics.

[37] Aronesty E (2013). Comparison of sequencing utility programs. Open Bioinforma. J..

[38] Nilsson RH (2010). An open source software package for automated extraction of ITS1 and ITS2 from fungal ITS sequences for use in high-throughput community assays and molecular ecology. Fungal Ecol..

[39] Edgar RC (2010). Search and clustering orders of magnitude faster than BLAST. Bioinformatics.

[40] Edgar RC (2013). UPARSE: highly accurate OTU sequences from microbial amplicon reads. Nat. Methods.

[41] Nilsson RH (2018). The UNITE database for molecular identification of fungi: handling dark taxa and parallel taxonomic classification. Nucleic Acids Res..

[42] Wright ES (2016). Using DECIPHER v2.0 to analyze big biological sequence data in R. R J..

[43] Murali A, Bhargava A, Wright ES (2018). IDTAXA: A novel approach for accurate taxonomic classification of microbiome sequences. Microbiome.

[44] (2020). NCBI BioProject.

[45] (2020). NCBI Sequence Read Archive.

[46] Sutela S, Poimala A, Vainio EJ (2019). Viruses of fungi and oomycetes in the soil environment. FEMS Microbiol. Ecol..

[47] Woodcroft BJ (2018). Genome-centric view of carbon processing in thawing permafrost. Nature.

[48] Mackelprang R (2018). Microbial community structure and functional potential in cultivated and native tallgrass prairie soils of the Midwestern United States. Front. Microbiol..

[49] Hervé V (2020). Phylogenomic analysis of 589 metagenome-assembled genomes encompassing all major prokaryotic lineages from the gut of higher termites. PeerJ.

[50] Clissmann F (2015). First insight into dead wood protistan diversity: a molecular sampling of bright-spored Myxomycetes (Amoebozoa, slime-moulds) in decaying beech logs. FEMS Microbiol. Ecol..

[51] Urich T (2008). Simultaneous assessment of soil microbial community structure and function through analysis of the meta-transcriptome. PLoS One.

[52] Geisen S (2015). Metatranscriptomic census of active protists in soils. ISME J..

[53] Tláskal V, Zrůstová P, Vrška T, Baldrian P (2017). Bacteria associated with decomposing dead wood in a natural temperate forest. FEMS Microbiol. Ecol..

[54] Moll J (2018). Bacteria inhabiting deadwood of 13 tree species reveal great heterogeneous distribution between sapwood and heartwood. Environ. Microbiol..

[55] Christofides SR, Hiscox J, Savoury M, Boddy L, Weightman AJ (2019). Fungal control of early-stage bacterial community development in decomposing wood. Fungal Ecol..

[56] Nayfach S (2021). A genomic catalog of Earth’s microbiomes. Nat. Biotechnol..

[57] Seibold S (2015). Experimental studies of dead-wood biodiversity — A review identifying global gaps in knowledge. Biol. Conserv..

