# Supplementary material for: Metagenomes, metatranscriptomes and microbiomes of naturally decomposing deadwood
Source: Sci Data. 2021 Aug 3;8:198. doi: 10.1038/s41597-021-00987-8 (PMC8333335; doi:10.1038/s41597-021-00987-8)

Supplementary Figure 1: Relative abundance of microbial taxa represented by A) ITS2 region and B) 16S rRNA gene in 25 samples of *F. sylvatica*. Samples are grouped based on the decomposition length in years. Only the most abundant phyla (or classes of *Proteobacteria*) are shown.

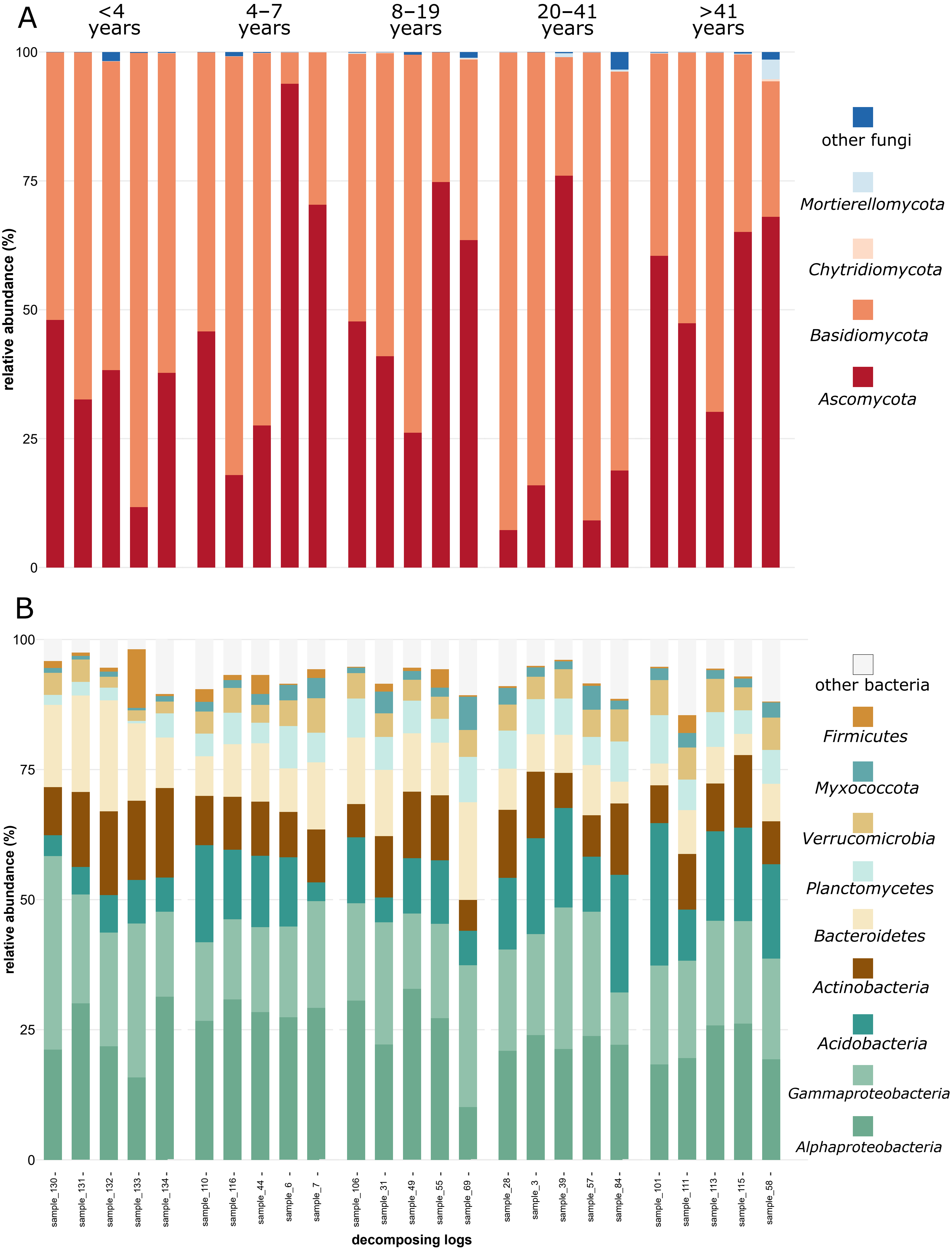

Supplement: Supplementary file 1 — Supplementary Figure 1 [file 41597_2021_987_MOESM1_ESM.pdf]
